# Electronic Health Record–Triggered Research Infrastructure Combining Real-world Electronic Health Record Data and Patient-Reported Outcomes to Detect Benefits, Risks, and Impact of Medication: Development Study

**DOI:** 10.2196/33250

**Published:** 2022-03-16

**Authors:** Karin Hek, Leàn Rolfes, Eugène P van Puijenbroek, Linda E Flinterman, Saskia Vorstenbosch, Liset van Dijk, Robert A Verheij

**Affiliations:** 1 Nivel, Netherlands Institute for Health Services Research Utrecht Netherlands; 2 Netherlands Pharmacovigilance Centre Lareb ’s-Hertogenbosch Netherlands; 3 Groningen Research Institute of Pharmacy Unit of PharmacoTherapy, - Epidemiology & -Economics University of Groningen Groningen Netherlands; 4 Tilburg School of Social and Behavioral Sciences (Tranzo) Tilburg University Tilburg Netherlands

**Keywords:** adverse drug reaction, general practice, patient-reported outcome, electronic health record, overactive bladder, research infrastructure, learning health systems

## Abstract

**Background:**

Real-world data from electronic health records (EHRs) represent a wealth of information for studying the benefits and risks of medical treatment. However, they are limited in scope and should be complemented by information from the patient perspective.

**Objective:**

The aim of this study is to develop an innovative research infrastructure that combines information from EHRs with patient experiences reported in questionnaires to monitor the risks and benefits of medical treatment.

**Methods:**

We focused on the treatment of overactive bladder (OAB) in general practice as a use case. To develop the Benefit, Risk, and Impact of Medication Monitor (BRIMM) infrastructure, we first performed a requirement analysis. BRIMM’s starting point is routinely recorded general practice EHR data that are sent to the Dutch Nivel Primary Care Database weekly. Patients with OAB were flagged weekly on the basis of diagnoses and prescriptions. They were invited subsequently for participation by their general practitioner (GP), via a trusted third party. Patients received a series of questionnaires on disease status, pharmacological and nonpharmacological treatments, adverse drug reactions, drug adherence, and quality of life. The questionnaires and a dedicated feedback portal were developed in collaboration with a patient association for pelvic-related diseases, *Bekkenbodem4All*. Participating patients and GPs received feedback. An expert meeting was organized to assess the strengths, weaknesses, opportunities, and threats of the new research infrastructure.

**Results:**

The BRIMM infrastructure was developed and implemented. In the Nivel Primary Care Database, 2933 patients with OAB from 27 general practices were flagged. GPs selected 1636 (55.78%) patients who were eligible for the study, of whom 295 (18.0% of eligible patients) completed the first questionnaire. A total of 288 (97.6%) patients consented to the linkage of their questionnaire data with their EHR data. According to experts, the strengths of the infrastructure were the linkage of patient-reported outcomes with EHR data, comparison of pharmacological and nonpharmacological treatments, flexibility of the infrastructure, and low registration burden for GPs. Methodological weaknesses, such as susceptibility to bias, patient selection, and low participation rates among GPs and patients, were seen as weaknesses and threats. Opportunities represent usefulness for policy makers and health professionals, conditional approval of medication, data linkage to other data sources, and feedback to patients.

**Conclusions:**

The BRIMM research infrastructure has the potential to assess the benefits and safety of (medical) treatment in real-life situations using a unique combination of EHRs and patient-reported outcomes. As patient involvement is an important aspect of the treatment process, generating knowledge from clinical and patient perspectives is valuable for health care providers, patients, and policy makers. The developed methodology can easily be applied to other treatments and health problems.

## Introduction

### Background

Electronic health records (EHRs) are increasingly used for the postmarketing surveillance of medicines, including information on prescription data, health care use, and morbidity [1,2]. However, EHRs lack information on personal significance or patients’ perspectives toward the use of medicines, including experienced adverse drug reactions (ADRs), and on patients’ health-related quality of life. Postmarketing surveillance should provide information about clinical and patient-reported outcomes (PROs). This allows insight into the benefit-risk balance of various treatments, including medication [3].

PROs provide in-depth insights into experiences and safety issues from the patients’ perspective. Such information is not routinely obtained in the premarketing phase of a medicine or during standard care. However, it would provide insight into how patients deal with their disease and treatment, and it can help patients and health care professionals in shared and informed decision-making. Thus, an infrastructure that combines clinical information from routine EHRs with patients’ experiences collected through questionnaires would allow the rapid generation of real-world information about the benefit-risk balance of various treatments, including medication, considering the perspectives of patients.

The infrastructure should be simple and reliable for patients and must have a limited administrative burden on the participating health care provider. Furthermore, it should be developed in such a way that it can be easily implemented for other diseases and treatments. Primary care is a suitable setting to develop such an infrastructure because most medications are prescribed in primary care. Moreover, in countries where primary care has a gatekeeper function, patients’ primary care EHR holds a complete record of morbidity and medication of a defined list of patients (most gatekeeping systems are also list systems, where general practitioner [GP] practices have a defined list of patients that they are supposed to care for), and thus, it provides an excellent opportunity to assess the benefit-risk balance of medication when complemented with PROs.

### Objective

This paper describes the requirements and the development of such a research infrastructure for the Dutch primary care setting called the Benefit, Risk, and Impact of Medication Monitor (BRIMM). We reflect on developing and using the BRIMM infrastructure as well as on its strengths, weaknesses, opportunities, and threats.

We chose the overactive bladder (OAB) as the use case to set up the infrastructure and assess its feasibility. OAB is a symptom-defined condition characterized by urinary urgency, usually with increased urinary frequency, waking up during the night to urinate, and sometimes with urgency incontinence. The high prevalence of OAB [3]; the increasing number of older adults; the negative effects of OAB on health-related quality of life [4]; and the presence of a relatively new medicine indicated for the treatment of OAB (mirabegron), which is under additional monitoring by regulatory authorities [5,6], makes OAB a suitable use case to develop and test this innovative way of collecting data for a benefit-risk registry in primary care.

## Methods

### Setting

BRIMM combines data from EHRs collected from general practices participating in the Nivel Primary Care Database (Nivel-PCD [7]) and PROs collected via questionnaires sent out with the Lareb Intensive Monitoring (LIM) system [8]. Nivel-PCD collects EHR data from almost 10% of the Dutch population (approximately 500 general practices, 1.8 million population). Data were collected since 1996 (from a small number of practices). Nivel-PCD contains data on consultations, morbidity, prescriptions, referrals, and clinical outcomes such as blood pressure measurements. Morbidity was recorded according to the International Classification of Primary Care version 1 (ICPC codes) used by the Dutch GPs. Prescription data were recorded using the Anatomical Therapeutic Chemical (ATC) classification. Nivel-PCD receives EHR data weekly from 350 practices, with more than 1 million listed people, allowing the identification of prevalent and incident OAB cases and following them over time. Data in Nivel-PCD are pseudonymized at the source (in the practices), leaving out directly identifying data such as names or addresses [9].

LIM is a tool to collect longitudinal PROs data; for example, on the occurrence of ADRs, coping, and impact on quality of life [10]. LIM was introduced in 2006 as a web-based intensive monitoring system to complement the spontaneous reporting of ADRs [8]. Patients received web-based questionnaires at different time points. The LIM system is flexible, as tailor-made questionnaires can be designed, which allows new questions to be added easily.

### Requirements

On the basis of these existing infrastructures, we began developing the BRIMM research infrastructure with a formulation of requirements. Following discussions with the project team, we formulated the infrastructure requirements shown in Textbox 1.

**Infrastructure requirements.**

**Versatile and flexible**

- Not limited to one drug or treatment

**Unobtrusive**

- Little or no interference with usual workflow
- Little extra work for general practitioners (GPs) or practice personnel
- Low threshold for patients to participate
- On the basis of existing infrastructures if possible

**Timely**

- Real-time or near real-time data collection
- Can be easily changed to monitor other treatments or diseases

**Useful**

- Contribute to better care (quality and efficiency)
- Generates information that is useful for GPs and patients during consultations
- Feedback loops to GPs
- Feedback loops to patients

**Legal**

- Compliant with the current data protection and privacy standards and legislation

This led us to set up the BRIMM infrastructure, which is described in the following sections.

### Design

**Figure 1:**
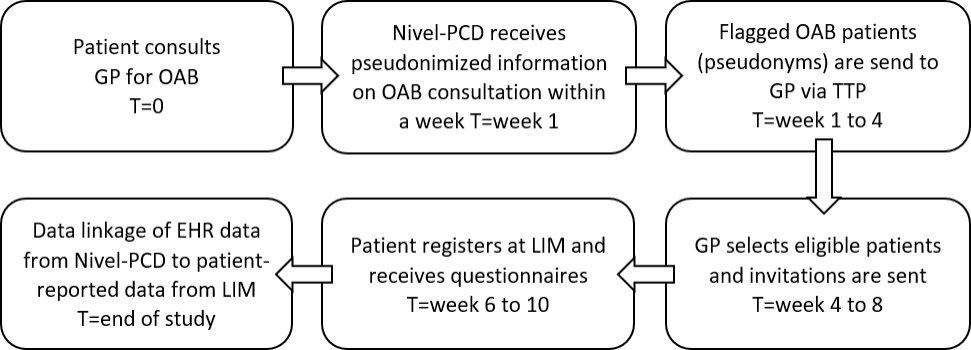
Benefit, Risk, and Impact of Medication Monitor workflow. T provides an estimate of the timing of the workflow. For this study, general practitioners received a monthly list of patients to check. EHR: electronic health record; GP: general practitioner; LIM: Lareb Intensive Monitoring; Nivel-PCD: Nivel Primary Care Database; OAB: overactive bladder; TTP: trusted third party.

Figure 1 shows a schematic overview of the workflow. GPs participating in Nivel-PCD were invited to participate in BRIMM. For participating practices, we used weekly data on prescriptions and morbidity to flag patients with prevalent and incident OAB. OAB cases were flagged on the basis of ICPC code U02 (urinary frequency/urgency) or U04 (urine incontinence) or a prescription with ATC code G04BD (drugs for urinary frequency and incontinence). Only patients aged ≥18 years were flagged, and those with prostate cancer (ICPC Y77) in their health history were excluded, as OAB is a frequent complication of prostate cancer. Prevalent cases were flagged based on information from the 3 months before study participation.

A study pseudonym was generated for each patient, which allowed for data linkage between the Nivel-PCD and LIM. Nivel-PCD holds one-way pseudonymized data only [9]; that is, Nivel cannot contact patients directly. As described elsewhere, in Nivel-PCD, it is possible via a separate extraction only accessible via a trusted third party (TTP) to link the pseudonyms with a patient identification number that is known only in the practices’ domain [9]. This allowed researchers to initially flag patients who could be eligible for the study and to let GPs subsequently decide whether they were eligible. For ineligible patients, the GP was asked to provide a reason for exclusion. GPs signed an agreement with the TTP, designating the TTP as the processor of the data. GPs provided the TTP with the eligible patient’s name and address, which were required to send an invitation letter. The TTP printed the letters and sent them to the patients on behalf of the GP. The invitation contained the patient’s study pseudonym.

If a patient decided to participate in BRIMM, they were enrolled in the LIM study, which aimed at collecting the PROs. The study pseudonym was entered by the patient and stored in LIM, allowing linkage between Nivel-PCD and PROs. Informed consent for study participation and data linkage was obtained during the registration process. Once registered, the patient received invitations to complete questionnaires at enrollment and every 3 months for a duration of 1 year.

This process of inviting patients was repeated monthly for participating GPs, flagging incident OAB cases. Patients’ name and address at the TTP were deleted 3 months after the end of patient recruitment (January 2020). A nonresponse analysis was performed using (pseudonymized) EHR data from flagged patients. GPs received a fee for each patient enrolled in the study.

### Data Collection

Patient questionnaires were developed by the project team in collaboration with an advisory board, representing key stakeholders of the patient association for pelvic-related diseases (Bekkenbodem4All), the Dutch College of General Practitioners (NHG), the Dutch Union of Urology, the Dutch Medicines Evaluation Board (MEB), a professor of pharmacy, and a health economist. Validated questionnaires were used if available. The questionnaire was only available on the web and covered the following topics. Topic A was asked in the first questionnaire, and topics B to F were included in all five questionnaires:

- Topic A: patient characteristics, including sex, year of birth, profession, education level, and socioeconomic position
- Topic B: start of OAB and contacts with health care providers for OAB
- Topic C: OAB-related symptoms (urogenital distress inventory-6 [11])
- Topic D: pharmacological and nonpharmacological treatments and treatment adherence using the Medication Adherence Rating Scale 5 [12] and the Exercise Adherence Rating Scale [13]
- Topic E: experienced ADRs
- Topic F: quality of life (EQ-5D-5L [14]) and bladder complaint–related (Incontinence Impact Questionnaire-7 [11])

### Privacy and Ethics Approval

Nivel and Lareb both maintain strict privacy protocols, which are also applied for this project. The email address of patients, which was required to send questionnaires, was provided by the patients upon participation in BRIMM. This information was encrypted and stored separately from the information collected in the questionnaires and EHRs. Nivel-PCD does not contain any patient-identifying information. Data were handled according to the General Data Protection Regulation to protect the privacy of participating patients and practices. The results cannot be traced back to individual persons, health care providers, or health care organizations.

This study has been approved according to the governance code of the Nivel-PCD (NZR-00316.050). The workflow was approved by the privacy committee of Nivel-PCD. The study protocol was assessed by the Medical Ethical Committee of Amsterdam Medical Center, location VUmc, who confirmed that the Medical Research Involving Human Subjects Act (in Dutch: Wet medisch-wetenschappelijk onderzoek met mensen [WMO]) does not apply to this study (#2017.506).

### Governance and Data Sharing

The governance structure of Nivel-PCD applies to BRIMM, which includes a steering committee, a privacy committee, and so-called chambers with representatives of health care providers. These chambers determine the use of data. For this study, an advisory board was set up, the composition of which has been described earlier. The advisory board participated actively in the project. In addition, we set up a patient panel to advise on the content of the questionnaires and patient feedback. The linked data resulting from this registry are available for use by parties other than Lareb and Nivel after approval by the governance structure of Nivel-PCD and Lareb.

### Feedback for Patients and Practices

For patients, two types of feedback were made available on their personal webpage where they filled in the questionnaires: (1) a PDF file of their filled questionnaire, which they could discuss with their health care provider, and (2) a graphic representation of some questions over time. The latter provided insights into changes in the patient’s OAB status, quality of life over time, and ADRs and experiences. Feedback in the graphic presentation was updated automatically after the questionnaire had been completed. The content was determined in collaboration with the patient association Bekkenbodem4All, and 10 patients provided feedback. Feedback to the patients was provided in (near) real time.

GPs received feedback on participating patients with OAB in their practice in comparison with other practices. Feedback for GPs provided insight into the number of participating patients, their prescribing habits, and the reported ADRs. Feedback to GPs was provided at the end of the study period. The feedback topics included health care utilization, adverse effects, adherence to treatment, and quality of life.

### Pilot Study

A pilot study was performed in 2 GP practices between January 2018 and August 2018 to test the feasibility of the infrastructure and to evaluate whether BRIMM could be improved from the perspective of GPs. Both practices were asked about their experiences with BRIMM in a semistructured telephone interview with the GP (practice 1) and practice nurse (practice 2).

### OAB Use Case

The pilot study was followed by a use case study in which GPs from the Nivel-PCD network were recruited. The GPs were invited by email and in groups of 20-30 practices. The invitation material was updated several times to test which material worked best. We emailed a flyer with a short description of the study, a link to a presentation with highlights, and an elaborate description of the study. Participating GPs received a frequently asked question document for practice personnel and a movie to be played in the GPs’ waiting room to introduce the study to their patients. A total of 27 GPs (including the 2 pilot practices) participated in the study. Patient nonresponse was described using descriptive statistics.

### Evaluative Strengths, Weaknesses, Opportunities, and Threats Analysis

In September 2018, Nivel and Lareb hosted a stakeholders meeting to inform stakeholders about BRIMM and retrieve their views on BRIMM. A total of 10 stakeholders attended the meeting representing a variety of institutions, including professional associations for GPs and pharmacists, the patient federation, pharmaceutical companies, pharmacovigilance center Lareb, the Medicines Evaluation Board, the national institutes on rational medicine use, and public health and environment. The BRIMM infrastructure and the results of the pilot study were presented at the meeting, after which 2 groups of stakeholders were formed and asked to evaluate the strengths, weaknesses, opportunities, and threats of BRIMM. The results of this evaluation are presented herein.

## Results

### Pilot Study

The patient recruitment route was successfully tested in the 2 practices that participated in the pilot study. A GP and a practice nurse checked their list of flagged patients monthly and provided the addresses of eligible patients. Both the GP and the practice nurse reported that the required time was as expected and that this was feasible in practice. They were also content with the information beforehand and the way and speed with which minor technical issues were handled, and they had no suggestions to further improve the patient recruitment route. They appreciated that the patients were referred to Nivel and Lareb for questions. Both practices would advise colleagues to participate in this project. The pilot study did not lead to any changes in the study design.

### OAB Use Case Results

A total of 27 GP practices participated in the study. In Nivel-PCD, 2933 patients with OAB were flagged. GPs selected 1636 patients (55.78%) who were eligible for the study, and 358 (21.88% of the eligible patients) registered for the study, of whom 295 (18.03% of the eligible patients) completed the first questionnaire. Practices recruited between 0 and 40 patients, with a mean of 10 (median 6, IQR 5-17) patients per practice. A total of 7 patients did not provide consent for data linkage; therefore, results that were calculated using EHR data were based on the 288 patients who provided consent for data linkage.

The main reason for excluding patients was no diagnosis of OAB (539/1297, 41.6% of exclusions; Table 1). Participating patients were, on average, slightly older (mean age 66, SD 13 years) and less often females (141/295, 47.8%) than those who were flagged (mean age 63, SD 19 years; 1741/2933, 59.36% females) and those who were invited (mean age 63, SD 18 years; 958/1636, 58.56% females; Table 2). The use of OAB medications was higher among participants, and participants had fewer chronic conditions than patients who were invited or flagged (Table 2).

We collected information on health care use, bladder complaints, and ADRs from 295 participants. The second questionnaire was completed by 163 (55.2%) patients. Of these, 102 (34.6%) patients completed all 5 questionnaires.

**Table 1:** Reasons for general practitioners to exclude flagged patients (n=1297).

Exclusion criteria	Patients, n (%)
No overactive bladder	539 (41.56)
Reason unknown	315 (24.29)
Cognitively or mentally unable	142 (10.95)
Moved or deceased	82 (6.32)
Cannot handle a personal computer	63 (4.86)
Treated by a urologist	53 (4.09)
Terminally ill or in hospital	46 (3.55)
Insufficient knowledge of the Dutch language	37 (2.85)
Other reasons	20 (1.54)

**Table 2:** Characteristics of the study population.

Characteristics			Flagged patients (n=2933)		Invited patients (n=1636)		Participating patients (n=295)^a^
Female, n (%)			1741 (59.36)		958 (58.56)		141 (47.77)
Age (years), mean (SD)			63.46 (19.05)		62.91 (17.89)		66.4 (12.91)
**Age (years), n (%)**							
	18-44	549 (18.72)		282 (17.24)		19 (6.42)	
	45-64	758 (25.84)		457 (27.93)		95 (32.32)	
	65-74	652 (22.23)		423 (25.86)		111 (37.58)	
	75-84	620 (21.14)		334 (20.42)		54 (18.32)	
	≥85	354 (12.07)		140 (8.56)		16 (5.43)	
Use of overactive bladder medication, n (%)^b^^,^^c^			640 (22.42)		392 (24.87)		68 (25.78)
**Number of chronic diseases, n (%)^b,d^**							
	0	405 (20.94)		203 (20.34)		31 (16.93)	
	1 or 2	725 (27.48)		421 (42.18)		80 (43.69)	
	≥3	804 (41.57)		374 (37.47)		72 (39.31)	

^a^Results of the 7 patients who did not give consent for data linkage between questionnaires and electronic health record data were not included in calculations on overactive bladder medication and number of chronic diseases.

^b^Information on medication use was available for 2854, 1576, and 264 patients, and information on chronic comorbidities was available for 1934, 998, and 183 patients.

^c^Overactive bladder medication was defined as medication with Anatomical Therapeutic Chemical code G04BD.

^d^The number of chronic comorbidities was based on a list of the 29 most common chronic diseases, including, for example, Chronic Obstructive Pulmonary Disease and cardiovascular disease [15].

### Results of Strengths, Weaknesses, Opportunities, and Threats Analysis

**Figure 2:**
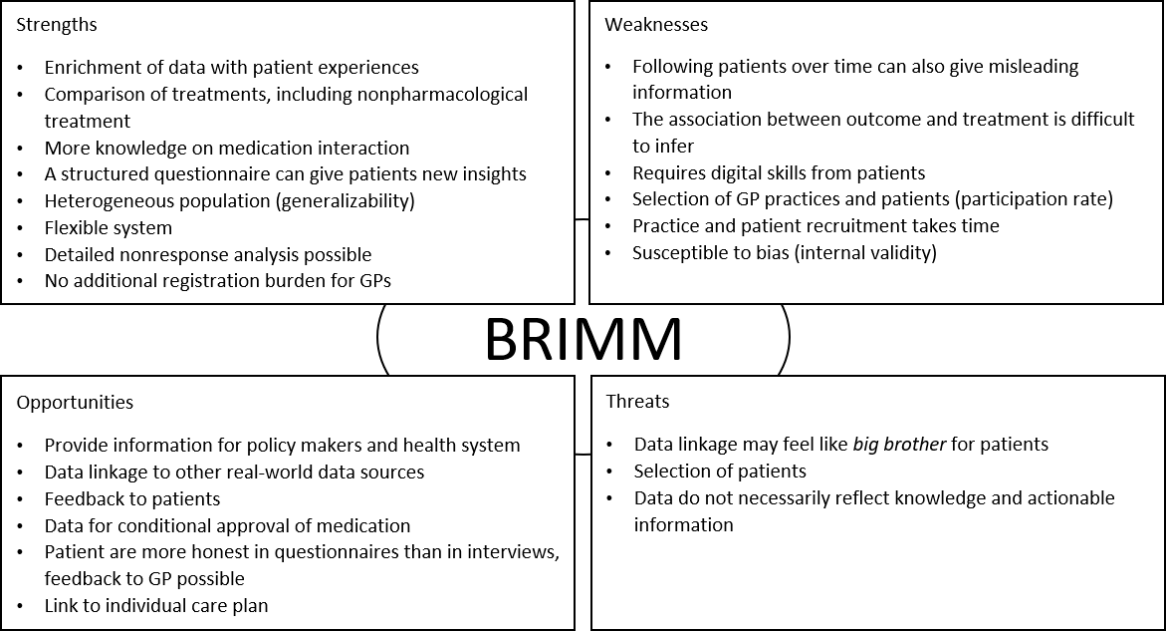
Summary of strengths, weaknesses, opportunities, and threats of the Benefit, Risk, and Impact of Medication Monitor infrastructure. BRIMM: Benefit, Risk, and Impact of Medication Monitor; GP: general practitioner.

Figure 2 provides a summary of the main findings of the Strengths, Weaknesses, Opportunities, and Threats analysis based on a discussion with stakeholders.

#### Strengths

A unique selling point of BRIMM is the combination of EHR data with patient experiences to compare pharmacological and nonpharmacological treatments. These data can be used to explore clinical research questions concerning specific diseases or treatments, patient perspectives, and economic evaluations. By including EHR data, BRIMM provides reliable information about concomitant medications and comorbidities. Moreover, by making efficient use of the EHR data during patient preselection, the final patient selection by the GP takes only a limited amount of time. Having EHR data for all patients in the preselection also enables a detailed nonresponse analysis and possible selection bias. Furthermore, the flexibility of BRIMM is an asset, and it can be adapted to other diseases or medications. Finally, a strength is the feedback that patients receive from their structured questionnaires. These may be valuable to patients, as it may provide a better understanding of their disease and the impact of treatment and support communication with the GP.

#### Weaknesses

A weakness that potentially affects study outcomes is that the association between outcomes and treatment is difficult to infer. Furthermore, this type of study is prone to selection bias for instance because of selective nonresponse by both GPs and patients. Both practice and patient recruitment requires time. This makes BRIMM less appropriate for answering acute, urgent research questions.

#### Opportunities

BRIMM may provide important information for policy makers and the health system and for individual (participating) patients and GPs in the form of feedback information and study outcomes. The data collected can be linked to other real-world data sources, and the outcomes of the study can be used to design individual patient care plans. In the future, BRIMM could be used for dedicated studies of drugs with conditional approval.

#### Threats

The stakeholders identified 3 possible threats. First, patients may distrust data linkage of EHR data with their questionnaire data, as they may feel that they are not in control of the nature of exchange of the data. Second, patient selection may affect the validity of the study outcomes. Finally, the amount of data collected does not automatically reflect the knowledge or actionable information.

## Discussion

### Overview

BRIMM is a unique research infrastructure that combines routinely recorded primary care EHRs with PROs to monitor the safety and benefits of medical treatment. Till date, studies have generally focused only on EHR data or PROs. By combining both types of data, we are able to provide a more complete picture of the patient characteristics and disease status (clinically and from a patient perspective), benefits and risks of treatments, drug use adherence, and quality of life. It also allows for a long-term follow-up of patients, which makes it possible to explore the long-term benefits and risks of treatments. BRIMM fits in with the trend of valorizing real-world databases [16,17].

BRIMM met our predefined criteria that it should be easy to use, timely, flexible, useful, and in accordance with legal requirements; however, we also encountered some practical challenges. Here, we reflect on the lessons learned from developing and testing the BRIMM research infrastructure that can be implemented for future studies combining EHR and PRO data.

### Lessons Learned

We extensively explored requirements for the BRIMM research infrastructure and composed general requirements to ensure the infrastructure would be widely usable, not only for the OAB use case. This means that this infrastructure can now be relatively easily implemented for other studies. For example, it took only a few months to prepare a second study on COVID-19 using the BRIMM method. For some studies, this might not be quick enough. Preparations, including development of questionnaire and ethical approval procedure, and GP and patient inclusion will take at least several months. Including patients for the BRIMM infrastructure only takes limited time for GPs. However, for any future studies, it should be considered that GPs have many other activities, and workload in general is an important barrier for participating in studies [18]. Thus, we pursued the GPs to participate by giving feedback, a fee, and highlighting the relevance of BRIMM in different forms (presentation, movie, and infographic). These efforts led to 27 GPs being included in the OAB use case. The study topic may also play an important role in the willingness of GPs to participate. GPs told us that OAB was not a topic of societal interest. On the contrary, an invitation for participation in a BRIMM study focused on patients with COVID-19 received interest of almost 100 general practices. Therefore, we recommend conducting an inventory among GP practices to attain their interest in the subject before the start of a next BRIMM-like study.

Stakeholders identified patients’ reluctance to consent to data linkage as a possible threat. However, this was not observed in this study, as 97.6% (288/295) of the patients consented to data linkage, which is in line with findings elsewhere [19,20]. For patient selection, it should be noted that BRIMM is set off by EHR data (ie, ICPC codes and ATC codes). The granularity of coding systems has been an issue in OAB use cases. The specificity of ICPC codes to include patients was limited; that is, there was no specific ICPC code for OAB. Therefore, GPs excluded a large group of initially flagged patients who did not appear to have OAB. In addition, we initially flagged some patients who should have been excluded because of prostate cancer. Apparently, prostate cancer had not been recorded in the EHR. This makes the infrastructure more suitable for diagnoses that can be flagged using a specific ICPC code or an ATC code. The more precise the initial flagging of patients, the less time consuming the efforts of GPs to check whether patients are indeed eligible.

Furthermore, the response rate of the patients was relatively low (295/1636, 18.03%). In general, response rates have declined over the past few years. Response rates may be increased by providing an incentive for patients to participate [21] and by using paper instead of web-based questionnaires [22,23]. However, web-based PRO collection also has several advantages, including lower costs and higher data quality [23,24].

Patient participation during the setup of the BRIMM research infrastructure was deemed important to ensure that the study benefits this patient group. However, involving patients in this study appeared to be a challenge. It took relatively much effort to receive feedback on the questionnaire. The feedback provided to the patients was hardly used by them, limiting the benefits for participating patients that we hoped for. Therefore, before conducting a study, it should be explored whether patients would like to receive feedback at all, and if so, what type of feedback they would like and how it should be made available to them. In addition, one can argue that providing feedback during the study may bias patients’ consecutive answers to questionnaires and, therefore, also the results. Moreover, this may be aimed for when providing actionable feedback. The infrastructure could then be regarded as an intervention as well as a research tool, along the lines of a learning health system [25-27]. However, we do not expect that the feedback in this study had biased the results, as the feedback was designed to provide a basic overview of adverse effects and bladder complaints and was hardly used. Depending on its aim, future studies should consider whether feedback should be provided during or at the end of the study.

The BRIMM research infrastructure can be used to investigate the benefits and risks of almost any treatment for which citizens consult their GP and may, for example, provide valuable information to support supplemental indications; that is, permission for medicines already on the market to be used for new indications, patient groups, or stages of disease [16,28,29]. However, to allow for the efficient use of resources, it is important to choose diseases or medicines with a high incidence, prevalence, or use in primary care to allow for the inclusion of a sufficient number of patients.

### Other Applications of the BRIMM Infrastructure

BRIMM’s unique combination of EHR and PRO data can also be used for other purposes than the monitoring of benefits and risks of medication, for example, to investigate the patient’s perspective regarding diseases and their treatments, such as the long-term effects of COVID-19 and the long-term effects of implants, for example, breast implants, or to study the effects of over-the-counter medication and home remedies for diseases. Furthermore, the BRIMM research infrastructure could be used to efficiently collect clinical and PRO data for clinical trials. Similar innovative methods to stimulate patient recruitment have been proposed before [30-32]. Finally, we will further analyze the data collected in the OAB use case to ensure the data are converted to actionable information.

The BRIMM research infrastructure was implemented in the Netherlands, a country with a primary care–oriented health care system and widespread use of relatively well-developed EHR systems in general practice. The infrastructure can also be implemented in other countries. Minimal requirements are a well-developed EHR system, digitally literate patient population, and sufficient quality of routinely recorded health data [33]. Furthermore, it is important to consider the position of GPs in the health care system, particularly with respect to the disease or treatment studied. Implementation in a primary care–oriented gatekeeper system, for example, and focusing on a disease that is primarily treated in primary care might be preferable. Within the Netherlands, the system can be implemented in other EHR databases and combined with PRO measures using the workflow described here.

### Conclusions

The BRIMM research infrastructure makes it possible to assess the benefits and safety of (medical) treatment in real-life health care situations using a unique combination of EHRs and PROs, and it does so in a nonobtrusive way, without causing much extra administrative burden for health care professionals. As patient involvement is an important aspect of the treatment process, generating knowledge from both the clinical and patient perspectives is valuable for both health care providers and patients.

BRIMM has significant methodological advantages to serve as a tool for postmarketing surveillance of drugs that require additional monitoring. In addition, it provides enhanced possibilities to create cohorts of patients to conduct large-scale comparative effectiveness research to accomplish the goals of a learning health system that supports patients, physicians, and policy makers in making informed decisions [25]. BRIMM can in principle be applied to any type of disease or medicine in primary care.

## Abbreviations

ADR: adverse drug reaction
ATC: Anatomical Therapeutic Chemical
BRIMM: Benefit, Risk, and Impact of Medication Monitor
EHR: electronic health record
GP: general practitioner
ICPC: International Classification of Primary Care
LIM: Lareb Intensive Monitoring
Nivel-PCD: Nivel Primary Care Database
OAB: overactive bladder
PRO: patient-reported outcome
TTP: trusted third party
